# Human beings as islands of stability: Monitoring body states using breath profiles

**DOI:** 10.1038/s41598-019-51417-0

**Published:** 2019-11-07

**Authors:** Kiran Sankar Maiti, Michael Lewton, Ernst Fill, Alexander Apolonski

**Affiliations:** 1 Max-Planck-Institut für Quantenoptik, Hans-Kopfermann-Straße 1, 85748 Garching, Germany; 2 Lehrstuhl für Experimental Physik, Ludwig-Maximilians-Universität München, Am Coulombwall 1, 85748 Garching, Germany; 3 Novosibirsk State University, 630090 Novosibirsk, Russia; 4 Institute of automation and electrometry SB RAS, 630090 Novosibirsk, Russia

**Keywords:** Diagnostic markers, Biophotonics

## Abstract

By checking the reproducibility of conventional mid-infrared Fourier spectroscopy of human breath in a small test study (15 individuals), we found that a set of volatile organic compounds (VOC) of the individual breath samples remains reproducible at least for 18 months. This set forms a unique individual’s “island of stability” (IOS) in a multidimensional VOC concentration space. The IOS stability can simultaneously be affected by various life effects as well as the onset of a disease. Reflecting the body state, they both should have different characteristics. Namely, they could be distinguished by different temporal profiles: In the case of life effects (beverage intake, physical or mental exercises, smoking etc.), there is a non-monotonic shift of the IOS position with the return to the steady state, whereas a progressing disease corresponds to a monotonic IOS shift. As a first step of proving these dependencies, we studied various life effects with the focus on the strength and characteristic time of the IOS shift. In general, our results support homeostasis on a long time scale of months, allostasis on scales of hours to weeks or until smoke quitting for smokers, as well as resilience in the case of recovery from a disease.

## Introduction

Breath can be considered as one of the human biofluids extracted from the body predominantly in gas phase. Medical diagnosis via breath has been an attractive option for many centuries^1–6^. This diagnostics has several unbeatable advantages over an analysis of blood^7,8^, specifically it is fully non-invasive, patient-friendly, and may be processed rapidly, allowing the collection of many samples per day. In this regard, a relevant question should be posed: why is breath analysis, as stated by A. Amann^6^, still in its infancy and not a routine tool for clinicians? The reason lies certainly not in an insufficient sensitivity for relevant substances, since e.g. gas chromatography mass spectrometry (GC-MS) allows the detection of hundreds of volatile organic compounds (VOCs) at the 10 pptv (part-per-trillion by volume) level.

The answer is definitely complex, at least threefold.

First, in addition to the sensitivity of molecular detection, data analysis and all the relevant existing standard operating procedures (SOPs) must be upgraded accordingly^3,9,10^. For example, the air quality in the experimental room (especially its degradation during the experiment) usually is not considered as well as the precise state of the breath donor.

Second, there are indications that GC-MS, ion mobility spectrometry (IMS), proton transfer reaction mass spectrometry (PTR-MS), selected ion flow tube mass spectrometry (SIFT-MS) and electrospray ionization mass spectrometry (ESI-MS)^11–15^ should be used cautiously in order to gather reliable information about the breath content. For example, the humidity in breath samples is not constant, causing problems in interpretation due to the dependence of the product ion distributions on humidity^15^ in all MS schemes. Also, these techniques have several limitations. Two examples of those: (a) several small alkanes and alkenes such as ethane, whose proton affinities are less than water, can’t be detected by PTR-MS, one of the most advanced and widely used MS techniques; (b) being developed for extraction and detection of large metabolites, ESI-MS has not demonstrated significant progress so far^16,17^ mostly because of the low signal-to-noise ratio (SNR) resulting from a low number of generated ions in gas phase. Another popular technique called electronic nose (e-nose)^18^, lacks physical and chemical control of the sample and therefore is so far not reproducible in different laboratories.

Third, most basic: because of insufficient progress in comparing healthy and diseased groups^19–21^, the common research strategy should be revised. As the first step in this direction, a study of the most stable group of the two must be performed, namely of a group of healthy individuals. So far, there is no understanding how stable are healthy individuals. If it turns out that this group has lack of stability, there is no sense to follow up the conventional strategy. But in the opposite case we will get a chance to increase the accuracy. Moreover, if the individual is found to be in general stable, the measured instability of his/her data can be considered as an indicator of the onset of a disease or as a health biomarker. The corresponding concept called island of stability (IOS) of individuals is described in the first chapter.

When we mention the absence of understanding of stability of a healthy individual, we consider an individual metabolic phenotype approach (IMP). In most cases, MS and nuclear magnetic resonance (NMR)^22,23^ were used to identify the stability of biofluids in liquid phase. The existence of an IMP has been under discussion for at least the last decade^22–24^, without an agreement in research communities. So far, the IMP activity delivered several promising, though not convincing results. They will be discussed together with our results about the IOS stability in the Discussion section. The results of IMP and IOS have direct relation to two main physiological paradigms, homeostasis and allostasis^25^. Homeostasis claims stability of a set of physiological parameters, allostasis - its variability and related resilience - its return to a steady state after termination of strong effects, like recovery from a disease.

Compared to MS or e-nose, optical (more specially, mid-infrared) spectroscopy represents the most fundamental technique for detection and identification of molecules. The detection does not need knowledge of additional parameters that have to be determined for the molecule in another experiment, except for those known from a quantum mechanical description. In order to measure the entire breath content, MS requires several techniques to be applied, whereas optical spectroscopy is capable of identifying all kinds of molecules in one experiment. Being conceptually better in terms of reliability and interpretability of results, it however meets severe technical problems. They can be condensed into the following two: (1) conventional laser-free Fourier-transform mid-infrared spectroscopy has both low sensitivity, of the order of ppmv (10^−6^) level, and even lower detectivity in case of breath study.

Detectivity can be defined as the detection sensitivity in presence of masking factors like water vapor. It means that there is no sense to improve the sensitivity alone further without improving the detectivity. Human breath contains a significant amount of water vapor (i.e. it has relative humidity approaching 100%). Therefore, the mid-infrared detectivity is severely degraded because of a large number of water vapor absorption bands in the informative spectral range; (2) the state-of-the art laser spectroscopy of different types being the most promising among the two, still (a) does not demonstrate higher SNR and detectivity in comparison to conventional laser-free Fourier spectroscopy, and (b) does not cover the necessary spectral range between 2.5 and 20 *μ*m (4000–500 cm^−1^) where biological molecules present in breath, have fundamental absorption bands^26^.

The main obstacle of laser spectroscopy is the lack of broadband mid-infrared lasers. Therefore, different nonlinear schemes are under development in several research groups aimed at efficient conversion of infrared radiation of the existing broadband femtosecond laser sources to high-quality mid-infrared supercontinuum. Recently, we reported on progress in development of a broadband high-dynamic range high-power laser mid-infrared spectrometer^27^. In short, more work needs to be done on this topic. Up to now, only 14 breath VOCs have been identified using different types of nonlinear laser schemes. However, this was not achieved in a single, unified experiment^28,29^. To note, the number of so far identified breath-related VOCs approaches one thousand^30^. In general, both laser and laser-free conventional Fourier spectroscopies have a long history and therefore are well-established techniques, verifiable at any procedure step. In a spectroscopic experiment, the steps are fast and do not need sophisticated pre- or post-processing except for water suppression. As we already pointed out, the water suppression procedure is unavoidable for each of the existing techniques for high quality breath analysis.

Here we show that conventional laser-free Fourier mid-infrared spectroscopy of breath, combined with significant water reduction exhibits significant information allowing to make conclusions about the validity of the IOS concept. In a preparatory experiment, we found and identified various VOCs in breath^31^ and shown in section 1 of Supplementary Information (SI). To simplify the terminology, we included carbon monoxide and carbon dioxide to VOCs. In the second set of experiments lasting 18 months and presented here, we conducted a study of a small healthy group by using the information about the VOCs identified in the preparatory experiment. Additionally, in a small set of snapshot studies we first, traced several effects present in ordinary human life and second, tried to distinguish short-time scale non-monotonic life effects from a long-time scale monotonic onset of a disease or recovery from a disease by using two examples.

In order to start snapshot or longitudinal breath studies of individuals with spectroscopy, relevant SOPs must first of all be established. The existing SOPs for any kind of bio-sampling imply steps of preparing, processing, and storing samples, with only rudimentary attention to the individual’s activities just before the breath sampling, listed on subject questionnaire. The idea to define an individual’s state as precisely as sampling and measuring SOP steps has already been addressed ten years ago^2,32^. This knowledge will in principle allow to take into account the current state of the individual under study and therefore to properly interpret the data. As an example, an elevated acetone level can be a signature of fasting^33^ or diabetes^34,35^. Though many attempts have been made to establish a set of SOPs for breath analysis, there is no agreement so far between researchers^9,10^. Therefore, the purpose of an advanced SOP aimed at providing maximum sensitivity and reliability of breath studies can be formulated as follows: an SOP should establish accurate procedures at each step of the study, with an individual’s state taken into account. It should be pointed out that the existing SOPs are mostly based on biochemical studies that deal with metabolites being different from those exploited in GC-MS, NMR and mid-infrared spectroscopy.

## Islands of Stability

**Figure 1:**
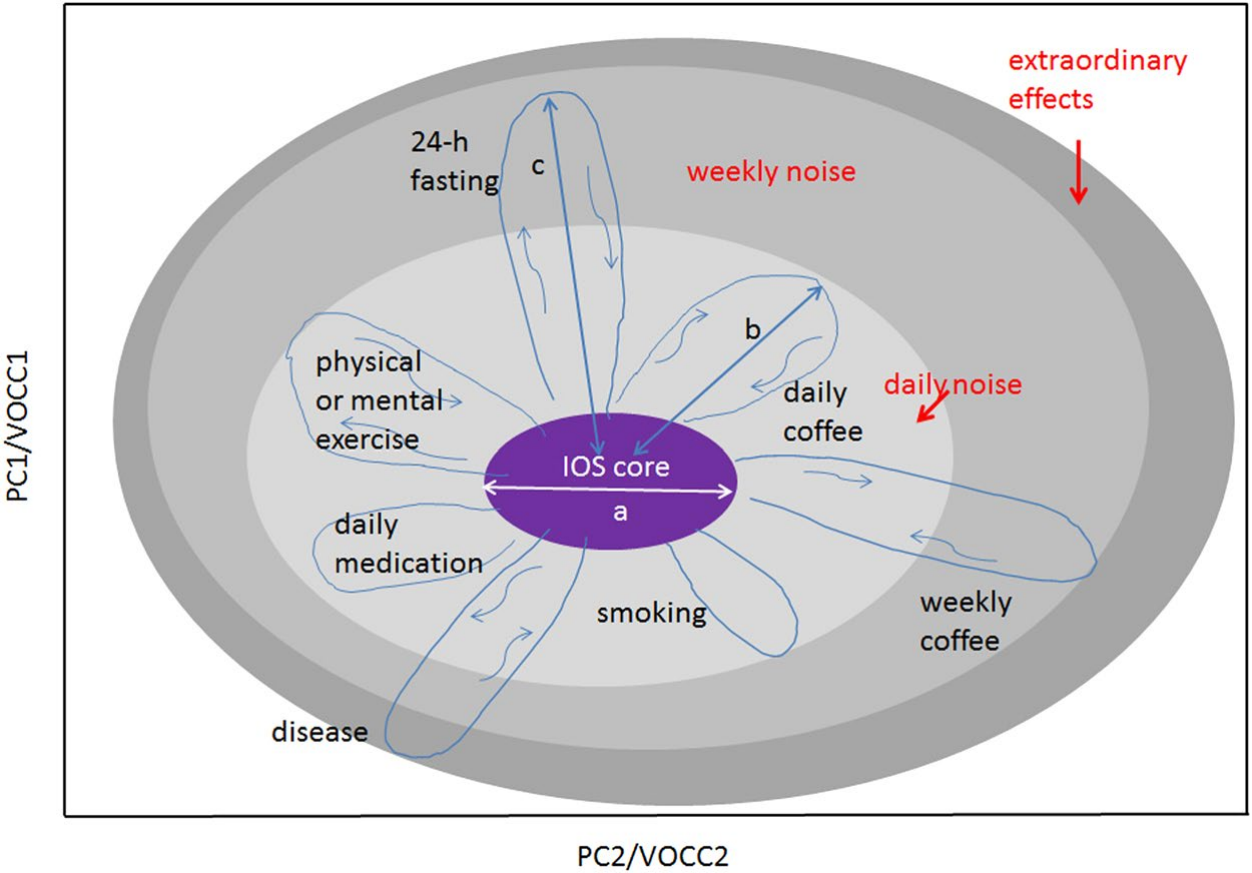
An illustration of the IOS concept for an individual. Any physiological parameters of the body can be presented on this graph. The space of representation can be blind (PC, canonical analysis) or show measurable variables like VOC concentrations (VOCC) as its axes. Shown: the light grey area represents several daily factors affecting the IOS core and increasing thus the measurable IOS size; medium grey area, factors affecting IOS on a weekly or monthly scale like fasting or coffee intake for rare coffee drinkers; dark grey area, extraordinary effects like strong stress or disease. There are two main scales making the concept quantifiable: the core size *a* and the strength of the effect *b*, *c* etc. In case of VOCC representation, scale parameters *a* and *b* are reduced to $\bar{n}$ and *δn*. The concept can be extended to many individuals. In this case, two other scales should be used: *a* and *l*, where *l* is the distance between the IOS cores. The higher the space dimensionality, the more cross sections can be found where any two persons will have *l* > *a*.

The concept of IOS is introduced in Fig. 1. The IOS can be defined in two ways: either it is a compact region in a multidimensional PCA plot (i.e. plot based on principal component analysis, usually with up to three components) determining the cloud of relevant data of an individual during the snapshot or longitudinal study, or else in a multidimensional plot exhibiting concentrations of important VOCs (the VOC-concentration representation, hereafter VOCC). As will be shown below, PCA should be used cautiously as it may carry information about uncontrolled parameters in the experiment. The IOS approach allows us to represent any physiological data of an individual as well as the effects affecting their variations, both in a quantifiable way. Unlike the IMP, the IOS term, as can be seen in the Results section, explicitly tells that a stable compact cloud of the healthy individual’s data does exist.

An individual can be described by his/her set of physiological parameters, reduced in our case to a multidimensional VOCC space in the following way: (${\bar{n}}_{1}\pm \delta {n}_{1},\cdots ,{\bar{n}}_{i}\pm \delta {n}_{i}$), with *δn*_*i*_ as concentration variations of i^*th*^ VOC during the longitudinal study. We call this set an IOS. A set of averaged values (${\bar{n}}_{1},\cdots ,{\bar{n}}_{i}$) or mean values, represents the IOS core, which is rather a mathematical description of the individual’s data of the highest precision. In ordinary life, the IOS of an individual represents a dressed state (or the noise-affected state containing all contributions to *δn*_*i*_), marked in grey in Fig. 1. The complexity of a dressed state can be illustrated by the situation when an individual makes several actions simultaneously or one just after another, like jogging after coffee intake^36^. In this case, the strength and duration of the effects listed in Table 1 could be different. Only experiment can answer the question whether extraordinary effects like disease have higher strength than ordinary life effects.

**Table 1:** Details about the experiments and their outcomes, with the reference to the corresponding figures illustrating them. Most of the experiments were performed with only few participants in order to get the first evidence about the strength and duration of the effects. The effect strength for VOCC space is defined as $\delta n/\bar{n}$. The data averaging was done only for smokers.

Effect	VOC	Effect strength, $\delta n/\bar{n}$	Duration, h
Cigarette for a heavy smoker, Fig. S2b	CO	1.5	1.5
IOS shift for a heavy smoker, Figs 2b and S2c	CO	6	constant
Coffee for a rare drinker, Fig. S1b	CO_2_	4,6	7
Coffee for rare drinker/moderate drinker, Fig. S1b	Blind, PCA	5/1	3/2
Physical exercise, Fig. S1a. Untrained/trained	CO_2_	4/1.5	3/2
Mental exercise, Fig. S1d	Isoprene/Acetone	10/1	2–5
Post-24-hour fast, Figs S1e and S3a	Isoprene/Acetone	2.3/13.5	10
Circadian variations, Figs S1f and S3a	Isoprene/Acetone	14.9/3.4	24
Alcohol (vodka, 1 drink), Fig. S1c	Ethanol	40	1.5
Normal food	Acetone	2.1	2
Viral disease, Fig. S4	Isoprene/Acetone	2.3/13	3 weeks
Immune mediated disease, Fig. S3b	Carbon monoxide/Isoprene/Acetone	2/2/1.5	4 weeks
Caffeine withdrawal plus absence of breakfast, Fig. S3a	Isoprene/Acetone	0.1/16	14

## Results

The traceable effects affecting daily, weekly and exceptional life cases that we studied are combined in Table 1 and illustrated in Figs S1–S3 (Section 2 of SI) in different parameter spaces. Table 1 contains specific VOCs, most sensitive to the effects, with the relative strength of the effects in terms of the scale parameters *a* (specifically $\bar{n}$) and *b* (*δn*) introduced in Fig. 1, and the duration of the effect.

The results of this study can be combined in three groups:

1. There is strong evidence that the IOSs exist, identified in this preliminary study for healthy individuals. Each IOS is uniquely placed in the VOCC space, with its unique size. Both the IOS core as well as the IOS itself stay stable for at least 18 months showing 20% deviations for the disease-free cases in that period. The only exception so far is methane for high methane emitters. For them, the natural data variations approach 100% (see Discussion section). Daily variations of VOC concentrations are of the same order of magnitude as the variations identified in the course of the longitudinal study. During the study, two participants contracted viral and immune mediated diseases with long time scale escape from IOS and the following return, that we unambiguously detected. Importantly, the individuals returned to their IOSs after recovering on a time scale of weeks.
2. For every individual, there are many ways to escape from his IOS e.g. by coffee ingestion, physical exercise, mental activity, smoking, fasting, alcohol intake etc., with the following return. The “journey time” and “travel distance” in these cases are different for each type of journey depending as well on the individual.
3. We recommend the following modifications of the SOPs: (a) the questionnaire should include strong effects listed in Table 1, and (b) direct breathing into the gas collecting system^32^ and the use of Tedlar bags provide comparable results if the storage time in the latter case is not longer than few days. Normal breathing provides reliable reproducible results (for details see Section 3 of SI).

## Discussion

Let us now focus on the finding of foremost importance, namely the evidence of compact and stable IOSs identified for all participants. The existence of an IOS is essential for several long-term targets: for realization of personalized medicine, for contributing to the problem of homeostasis and allostasis^25^ and for establishing early disease detection. As an illustration for the latter, a monotonic IOS shift in VOCC space can be considered for that purpose.

**Figure 2:**
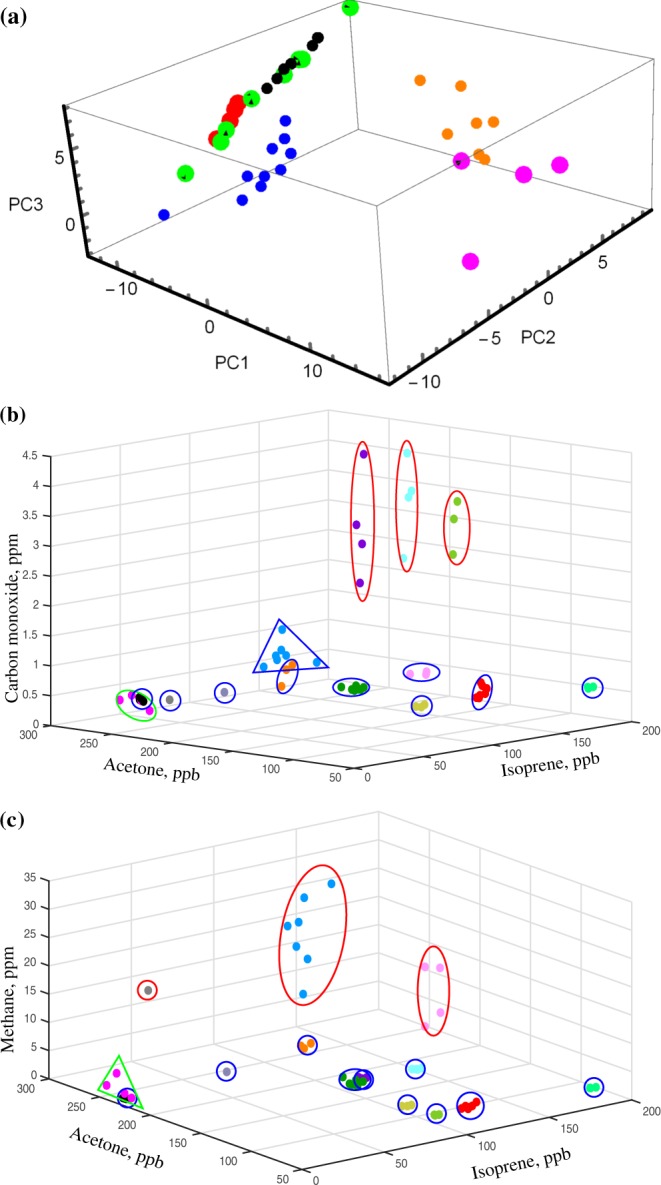
An illustration of 3D blind PCA (**a**) and VOCC data representation (**b**,**c**) collected during 18 months. (**a**) Magenta and orange points show high and medium methane emitters, blue - smokers, green, red and black - the rest of the volunteers. The corresponding p-parameter for high and medium methane emitters vs low emitters is 3 × 10^−19^, for smokers and nonsmokers: 8 × 10^−5^. Among 2D PCA representations, only a PC3/PC2 plot allows to separate smokers and nonsmokers with high selectivity, p < 10^−11^. (**b**) Large red ovals mark smokers, with clear separation from non-smokers. (**c**) Note larger methane variation of high methane emitters in comparison to medium and low emitters.

As we already pointed out, there are still debates about stability of the individual’s biofluids over extended periods of time. It turned out that for several biofluids, their stability in the body can last for years^25,37^, though with a decreasing probability of the individual identification. First studies of healthy cohort variations backdated to 1999, but neither there^38^ nor in further publications^9,39^, the individual’s breath state had been discussed on the time scale of more than a few days. For breath, stability of several small metabolites within 9 days was confirmed by means of GC-MS^39^. The overall recognition score of 11 healthy individuals achieved 76%, meaning the probability to unambiguously link the non-assigned experimental point to one or another individual. In the current study, we found that the individual’s breath content can remain stable over the period of 18 months even without having conventional CO_2_ control called capnography, during the sampling procedure (for details, see Methods). The overall recognition score in our case, withdrawn from Figs 2b,c and S5, is equal to 100%.

From VOCs we have measured so far, methane on a time scale of months demonstrates highest instability. Though the average methane emission can stay stable for up to 3 years^40,41^, irregular month-to-month variations by a factor of two for high emitters have been shown^42,43^. In our case of high emitters, the strength parameter $\delta n/\bar{n}$ deviates a factor of two less. We explain the difference by higher measurement accuracy in our case, intrinsic to the spectroscopy in comparison to GC-MS. In general, all microbial-derived molecules have highly variable levels in all individuals^44^. The origin of the instability is still unknown. Two aspects of the upgraded SOP relevant to the precise methane measurement can be found in Section 3 of SI.

Almost all the effects collected in Table 1 were already studied for biofluids (in liquid and gas phases) and reported in journals devoted to health, diet, sport and specific diseases. The new points here are the following: (1) to our surprise, so far breath was not measured *after* but only during physical exercise^45^. This point is important for the questionnaire that we attribute to SOP; (2) similarly, the decay of the elevated acetone concentration *after* fasting was not measured^46^; (3) we also have not found a 24-hour breath study; (4) the smoking effect in our observations is much stronger than previously reported^47–51^, unambiguously splitting smokers and nonsmokers (Fig. S2); (5) the metabolites we identified as most sensitive for life effects in Table 1 are different from those exploited in the publications. Otherwise, our results support previous publications regarding physical exercise^36,45,52–54^, coffee^36^ and alcohol^55^ intake, mental activity^36^, circadian variations^56^, food^34,36,46^ and fasting^57,58^. An essential advantage of our study is that here we can compare the results of different effects by using the same rule, see Table 1, with the same accuracy along the study period. Namely, by exploiting the IOS scale parameter *a* (introduced in Fig. 1), we can compare the strength of the effects shown as *b*, *c* etc. affecting the escape. For example, the “escape” effects of fasting or mental exercise can exceed 10 IOS units (i.e. $10\times \delta n/\bar{n}$), being thus considered as strong ones. In contrary, the postprangial “escape” of 2 IOS units can be considered as a weak effect.

As can be seen in Fig. 2b, smoking in terms of carbon monoxide leads to a permanent shift of the IOS. A similar behaviour was identified earlier for acetonitrile^50^. These data support allostasis i.e. temporal adaptation of the human body to external factors. We expect a similar IOS shift for individuals with chronic disease taking medications. For 2 detected disease cases in our study, return to the IOS after recovering supports resilience^25^. Resilience can act at any time scale in cases of disease or quitting smoking. Other effects studied in this test have durations up to 10 hours suggesting thus short-term allostasis. On a long time scale of months, our data in general support homeostasis. Natural methane variation on different time scales identified here and in^43^ is a good illustration of long-term homeostasis in presence of daily-to-monthly variations of unknown origin^41^. Summarizing this short phenomenological part, we see no contradiction between the models of physiological regulations discussed above because each of them has its own niche and together they explain variations of human states as functions of external factors and various body conditions revealed at different time scales.

A personal passport containing the IOS data averaged over some period could help in the strategy aimed at early detection of abnormalities in the body. The strategy implies that a new measurement point (${n}_{1},\cdots ,{n}_{i}$) of an individual should be compared with the position of his/her IOS core identified earlier during the passport establishment. In the case of a repetitive shift of the IOS position after several consequent measurements, an abnormality should be considered. The realization of the strategy would be possible only if clinicians accept it.

For the statistics we gathered and for the dimensionality of the parameter space we have at the moment at our disposal (see Section 1 of SI and Table 2), the intrapersonal variations *δn*_*i*_ in average are definitely smaller than the interpersonal separation, if we use conventional terms, see for illustration Fig. 2b,c and Section 4 of SI. Importantly, this is not the case for the PCA representation in Fig. 2a. In case of blood spectroscopy, usually only a few-dimensional space from absorption spectra is available for the principal component or variance analyses leading to comparable values of intrapersonal variations and interpersonal separation. In both cases of blood and breath, a relevant question arises whether these status quos will be valid for larger statistics. The expected answer is obviously no, unless we increase the space dimensionality. In general, the higher the parameter space dimensionality the more VOCC projections where the separation between any two individuals will be larger than their IOSs.

**Table 2:** A details about volunteers and in experiments they have participated.

Number of volunteers	15
Body mass index, average	25
Male/female; age range	8/6; 30–77
Number of probes	73
Number of volunteers/number of experiments they participated	3/7; 6/4; 3/3; 1/2; 2/1
Number of methane emitters; ratio to the total number of volunteers	5; 0.35
Period of study	September 2017–August 2018
Number of contracted/detected diseases during the study	2/2
Number of voluntees using direct breathing	4
Number of volunteers using Tedlar bags	15

As the carbon monoxide concentration in breath can be affected by several effects like smoking or fasting or immune mediated disease (Figs S2–S4) and carbon monoxide has low solubility in blood, it makes sense to compare it with the corresponding blood level. In the case of smoking, we see good correlation between the two (see Section 5 of SI). Cigarette smoke contains hundreds of VOCs, and many compounds other than carbon monoxide can also be used for comparison of smokers and nonsmokers^50,51^. As an example, acetonitrile in smokers was found at a level increased by a factor of 1.5^50^ compared to a factor of 6 for carbon monoxide (see Fig. S2 and Section 5 of SI).

In conclusion, we point out the following:

Under the current absence of broadband mid-infrared laser spectrometers of the pptv sensitivity and detectivity levels, a conventional upgraded Fourier spectrometer of 50 ppbv detectivity for small metabolites used in this study, is a promising tool for human breath analysis. The results of our test experiments focused on feasibility of monitoring several ordinary effects affecting humans, support the literature data, with few exceptions discussed above. During a 18-month test experiment, all the healthy participants demonstrate stable sets of their unique breath content (stable IOSs), with variations of less than 20%, whereas methane exhibits 100% variations.

## Methods

We used a Fourier spectrometer (Bruker Vertex 70) operating in a spectral range of 500–4000 cm^−1^, in conjunction with a 4 meter, 2 liter multi-pass “White cell” that collects breath for the measurement, and a liquid nitrogen cooled MCT detector. For all measurements, 0.5 cm^−1^ spectral resolution was used, with averaging of 100 scans. The resulting sensitivity of the spectrometer achieved 50 ppbv for several VOCs^31^. For revealing the dependence of the spread of collected data on the detection noise, the MCT detector was used at room temperature for comparison. The data collected in the latter case, demonstrated significant spread in PCA space, up to a factor of 10. Breath comes into the cell via a gas system containing a water condenser and a needle valve providing breath flow control through the condenser in order to establish a time interval necessary to efficiently freeze the water vapor from breath^31^. During the measurements, the spectrometer was flushed with dry nitrogen in order to remove water vapor from the optical system.

All measurements were done in one laboratory at +20 °C and humidity between 35 and 60%. Most of the breath sampling (except circadian variations) has been carried out during the active part of the day, between 7 am and 8 pm. Details about volunteers and number of probes collected in the study are combined in Table 2. The Ludwig Maximilians University of Munich’s ethics committee reviewed the study protocol and granted exemption from approval. All volunteers being a part of our research group, prior to study signed an informed consent form. The first group of four healthy volunteers agreed for a longitudinal study, used direct breathing into the spectrometer measurement cell through the mouthpiece (Eppendorf). It took about 5 minutes to fill the gas cell up to 0.5 bar pressure, with approximately 25 exhalations. Another group of 11 volunteers (men, women of age between 30 and 50 years) used only Tedlar bags (Sigma Aldrich), with the following measurements through the same gas and optical scheme as the first group. Tedlar bag is a good practical choice for extensive studies^59^. With the sensitivity of our measurement system, we found only slight degradation of the Tedlar bag content within days, mostly visible for carbon dioxide. One and two liter Tedlar bags were used only once. In case of the 1 liter Tedlar bag, it took about 1 minute to fill it with 4–5 breaths. For each measurement of both groups, the whole gas system was cleaned several minutes by dry air and evacuated down to 10^−4^ mbar. Tedlar bags were also used for estimating the error bars of the measurements. To this aim, a volunteer filled several Tedlar bags in the same time by using a simple T-shaped gas distribution pipe system. The content of the Tedlar bags was then analyzed as independent samples, with further comparison of the results. The resulting error bar was evaluated as 5%. In the case of the fasting and circadian rhythm experiments, Tedlar bags were stored one week after sampling at 4 °C before the measurement.

For experiments aimed at defining the value of the daily or weekly noise via coffee intake, physical and mental exercises, volunteers of two types were chosen: with expectedly high and low levels of reaction. One volunteer does not do regular physical exercise and drinks coffee irregularly, approximately 1 cup per 2 weeks (a rare drinker). Another volunteer was chosen as a heavy coffee drinker (5 cups per day). Standard cups of coffee from a coffee machine were used before the experiment and the resulting data were compared without any attention to the individual’s physical state or previous meal intake. A mental exercise included one volunter who played 5 chess games during one hour (blitz, 5-min for a player) via internet on a chess website (chessbase.com with his and the partners’ ratings around 1800). Two persons of different training stages participated in physical exercise study. One participated twice, with 30-minute outdoor and indoor run. Another, more trained person made intense 30-minute outdoor bicycle trips (approximately 12 km along a gravel road). A 24-hour fasting experiment and a 27-hour experiment monitoring circadian variations implied usual life style of the volunteers. Three smokers were studied: two moderate smokers (female, 5 cigarettes per day) and one heavy smoker (male, 20 cigarettes per day). To present and analyze the data, we used two approaches: PCA in combination with ANOVA for quantitative conclusions about data clustering, and visual inspection of the absorption spectra for VOCC representation. For the former, a code written in Mathematica was used. For the latter, we used Matlab allowing for the correction of the baseline distortions caused by the liquid-nitrogen detector at high amplification. As the experiments were performed in several sets, and each set was made within one day, the data within a set were considered as most trustworthy whereas the data of different sets were considered with precaution.

## Supplementary information

Human beings as islands of stability. A breath study
