# Supplementary material for: Human beings as islands of stability: Monitoring body states using breath profiles
Source: Sci Rep. 2019 Nov 7;9:16167. doi: 10.1038/s41598-019-51417-0 (PMC6838060; doi:10.1038/s41598-019-51417-0)
Supplement: Supplementary file 1 — Human beings as islands of stability. A breath study [file 41598_2019_51417_MOESM1_ESM.pdf]

Human beings as islands of stability: Monitoring body states using breath profiles

Supplementary information

Kiran Sankar Maiti[1,2], Michael Lewton[1], Ernst Fill[1,2], and Alexander Apolonski[1−4]
[1]Max-Planck-Institut für Quantenoptik, Hans-Kopfermann-Straße 1, 85748 Garching, Germany
[2]Lehrstuhl für Experimental Physik, Ludwig-Maximilians-Universität München, Am Coulombwall 1, 85748 Garching, Germany
[3]Novosibirsk State University, 630090 Novosibirsk, Russia
[4]Institute of automation and electrometry SB RAS, 630090 Novosibirsk, Russia
Apolonskiy@lmu.de

A list of sections referred in the main text:

1. The measured and identified molecules.
2. Illustration of the life effects combined in Table I.
3. Steps upgrading the SOP.
4. The proof of 100% score of the data shown in Figure 1c.
5. Relation of CO in blood and breath

1 The measured and identified molecules.

In our study, we identified 8 molecules: carbon dioxide, carbon monoxide, isoprene, acetone, methane, ethane, ethyl ester of propionic acid, propyl ester of propionic acid and aldehydes as a family of molecules having the characteristic absorption peaks in the range 1680-1740 cm^{-1}. We also observed several non-identified spectral features. The reason for that is the following: a molecular identification is a complex process because of 1) the masking effects of water[1] and carbon dioxide and 2) an overlap of absorption spectra of different VOCs of unknown concentrations. In our case, the identification includes several consequent steps. Firstly, the molecule candidates are considered from the NIST library[2] due to their characteristic spectral features observed in the experiment. Secondly, the candidates from the NIST list become selected after comparing them with the compendium of VOCs.[3] Thirdly, in case of several candidates or/and because of insufficient accuracy of the NIST data, they become available as substances and measured in our spectrometer under the same conditions as breath samples. In such a way, for example, derivatives of propionic acid were unambiguously identified.

2 An illustration of the life effects combined in Table I.

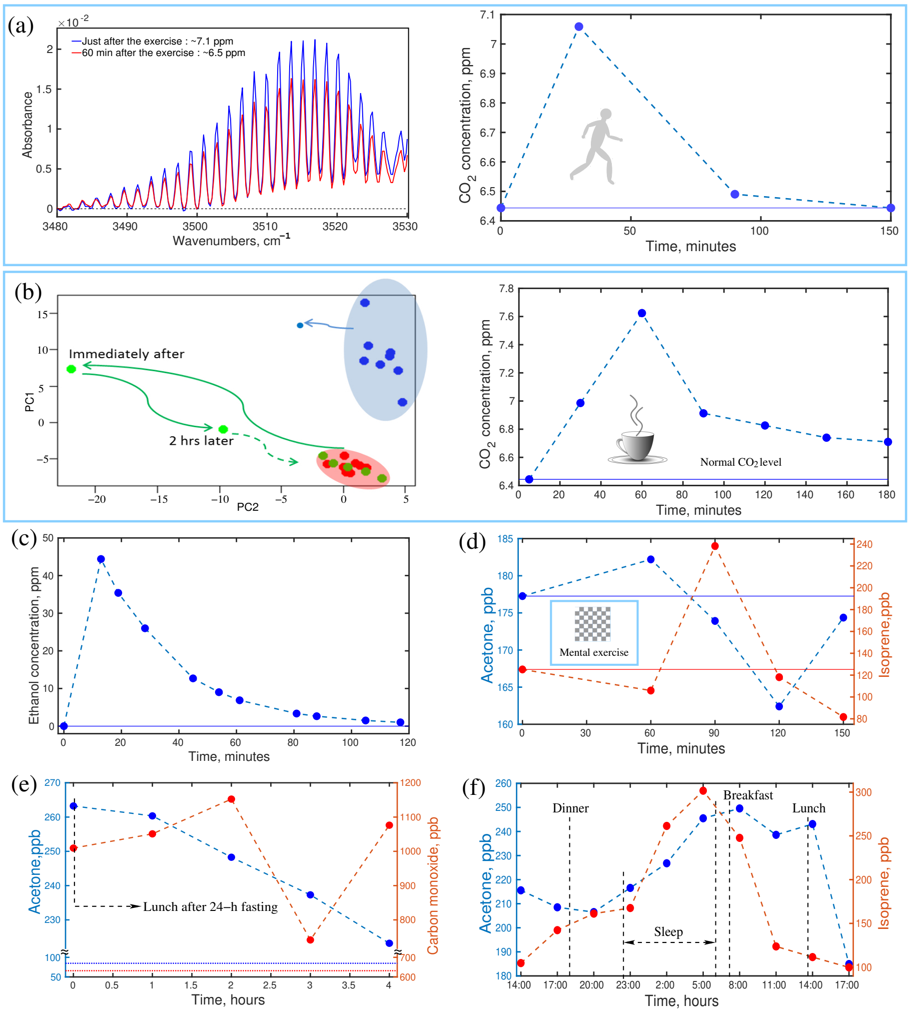

Figure S 1: An illustration of the effects affecting the IOS. (a): physical exercise (jogging); left, two absorbance traces of the CO_2 absorption band at different moments after the exercise; right, CO_2 concentration in time (right). The left point is taken just before the exercise. (b): coffee intake; PCA representation of the IOS escape and return (left) for a rare coffee drinker (green) and a moderate drinker (blue), and CO_2 concentration of a rare coffee drinker in time (right). Note that the effect reaches its maximum 1 hour after the intake and lasts up to 7 hours (evaluation). (c): alcohol intake (1 drink of vodka); the left point corresponds to the intake. Similar to coffee, the maximum of the ethanol concentration in breath is achieved with a delay, in this case after 15 minutes of intake; (d) mental exercise (chess play); horizontal lines show the steady level of the corresponding VOCs. (e): variations of isoprene and CO after the first meal ending a 24-hour fast. Horizontal lines: steady state levels of the corresponding VOCs. (f): acetone and isoprene circadian variations. The size of the experimental points in all graphs corresponds to the error bars. The dash lines in all Figures are used to connect the experimental points separated in time.

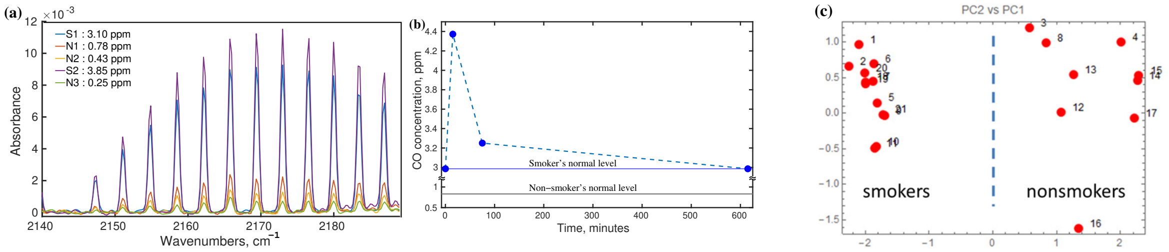

Figure S 2: Comparison of smokers and nonsmokers via CO measurement in breath. (a): absorption CO spectra of smokers S (magenta, heavy smoker and blue, moderate smokers) and nonsmokers N (green, yellow and orange). Each spectrum structure corresponds to several vibrational CO modes visible as peaks, leading thus to the high resulting SNR. (b): relative concentration of CO of a heavy smoker before the cigarette (left point) and after; the right point: after the nonsmoking night (10 hours). Low blue and black lines: average steady-state level of CO concentration for smokers and nonsmokers, correspondingly. (c): PCA +ANOVA representation, $p<10^{-8}$. The numbers near to experimental points show the order of consequent measurements during one day. A vertical dashed line serves for visualization of the two groups.

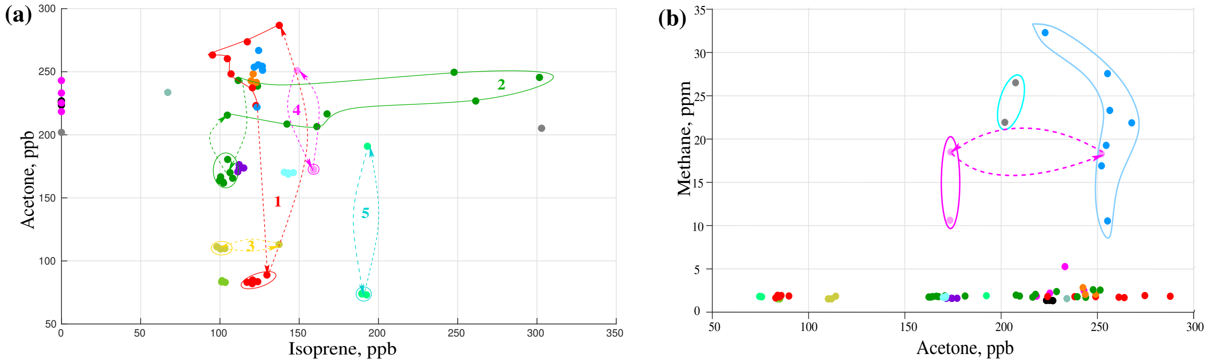

Figure S 3: 2D illustration of several effects affecting the IOS on isoprene-acetone (a) and acetone-methane (b) plots. Identical colors in plots correspond to the same volunteers. Dashed lines used for visualization of the loops, show the trajectory parts not measured in the experiment. 1 (red): 27-hour fasting; 2 (green) and 3 (olive): circadian variations, a full cycle (green) and partial (olive; the right point corresponds to 7am and 3 left points - to the period 12 am–3 pm); 4 (magenta): effect of disease (between two contracted diseases within one month). Note that the bottom shaded circle in (a) contains two overlapped points measured in non-adjacent months; 5 (cyan): 10-hour caffeine withdrawal and absence of breakfast for a heavy coffee drinker. Note the following: correspondence of loops 1 and 5, 2 and 3 in (a); significant variations of the points of a medium methane emitter (blue) along the methane and acetone axes; grey points significantly separated in (a) and corresponding to isoprene concentrations 0 and 300 *ppb* are close in plot (b), at acetone concentration around 200 *ppb* ; the escape of the IOS core of a medium methane emitter during the viral disease (magenta) visible in both plots; a significant number of low-methane emitters in plot (b) and several low-isoprene emitters in (a).

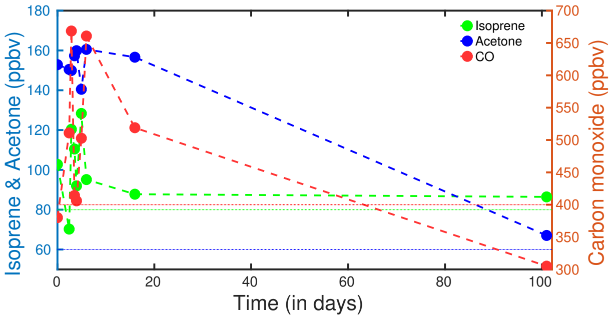

Figure S 4: The case of immune mediated disease. The horizontal green, red and blue lines correspond to the $\overline{n}_i$ values of the IOS core. The period of the first eight days on the plot corresponds to the active disease phase.

3 The SOP upgrade.

In addition to the life effects that should be, in our opinion, a part of SOP (questionnaire), two essential technical SOP aspects have to be considered.

The main reason for the breathing stability in our experiments in the absence of conventional CO_2 control is reproducible breathing by the volunteers ("the best effort" approach). For longitudinal studies, we found it as a practical solution. Our confidence is based on direct comparison of two breathing procedures that we compared: a) direct breathing into Tedlar bag and into the gas system[1] and b) normal and alveolar breathing. The latter point means that in the case of alveolar breath the contribution of ambient air will be minimized. We found that a) there is strong correlation between normal and alveolar breathing and b) the influence of ambient air can be reduced in several ways.[4] In our case, we a) used similar conditions in the laboratory (fresh air during the measurement campaign) and b) measured the concentrations of VOCs under study in air. For example, we found that all VOCs from the list in section 1 of SI were absent, bearing in mind the current detection sensitivity.

For successful detection of VOCs of lower concentration than in ambient air, it is critically important that the normalized (i.e. relative to that in ambient air) VOC concentration under study should be higher than the error bar of the measurement. This calls for another challenge in terms of experimental accuracy needed for such a detection. The challenge gives spectroscopy a good chance for success in comparison to other techniques.[5–9] At least, a minimal number of pre- and post-processing steps in case of spectroscopy accompanied by the control of VOC concentration at each measurement step, supports this argument.[4]

4 The proof of 100% score of Figure 2.

The inset of Fig. S5 shows the suspicious case of the two sets of data in green triangular. As one can see, there is no overlap between the two IOS sets. Another proof procedure available in Matlab, contains fast arbitrary rotation of the 3D plot in the same scales as in Fig. 2b, with the corresponding visual inspection. By applying it at different angles of view, we proved an unambiguous separation of any two individuals in our test group. The ultimate proof that can be considered for large statistics includes high-dimensional VOCC space, with a similar procedure described just before, for different sets of 3D projections.

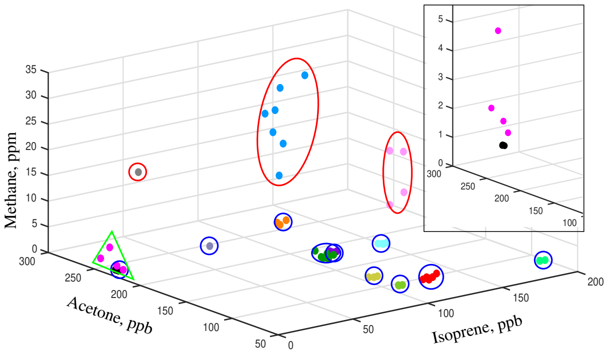

Figure S 5: Figure 2 of the main text with the zoomed in part from green trangular.

5 CO level in blood and breath of smokers.

In general, a comparison of blood (as well as other biofluids) content in a liquid and gas phases, being either biochemical, spectroscopic (mid-infrared or nuclear magnetic resonance[8, 9]), or mass-spectrometric would be very interesting. In our opinion, these phases are complementary in terms of available information. To illustrate this for small metabolites circulating in blood, we can mention poorly soluble substances like carbon monoxide (CO). In blood, it propagates in two forms: in a dissolved form and by using a carrier, namely hemoglobin forming thus carboxyhemoglobin (COHb).[10] In case of smokers, in addition to the endogenous source of CO in the body, much higher amount of CO comes into blood via lungs. There is always a dynamic equilibrium of CO circulating in blood in both forms, and CO in breath due to the back release of CO from blood into exhaled air in alveoli, different for dissolved CO and COHb. An evaluation based on data in Fig. 2 shows that in case of 1% of CO in breath, approximately half of the total amount of CO in blood (corresponding already to 6%)[10] is dissolved and can't be determined via direct measurement. MS techniques, spectroscopy or biochemistry are capable of measuring only the second CO contribution, namely COHb concentration. It means that in order to precisely determine the amount of CO in blood, one has to use both approaches: measurement of decays of COHb concentration in blood[11] and CO concentration in breath. It is agreed that it takes from 6 to 24 hours after the cigarette intake until the CO concentration in blood will come to norm. In the experiment we see that even after 10 hours after the cigarette, the CO level in the heavy smoker's breath (and, correspondingly, in blood) stays 6 times higher than that of a non-smoker (Fig. 2b). A simple estimation of the CO decay from Fig. 2b gives 10 days for the total return to norm. This discrepancy calls for an additional strong mechanism of CO evolution in blood in the case of its extra and repetitive intake with a cigarette, like accumulation in tissues with further release.

References

[1] Maiti, K. S., Lewton, M., Fill, E. & Apolonski, A. Sensitive spectroscopic breath analysis by water condensation. *J. Breath Res.* **12**, 046003 (2018).

[2] NIST Chemistry WebBook. `https://webbook.nist.gov/chemistry/`.

[3] de L. Costello, B. *et al.* A review of the volatiles from the healthy human body. *J. Breath Res.* **8**, 014001 (2014).

[4] Miekisch, W., Schubert, J. K. & Noeldge-Schomburg, G. F. Diagnostic potential of breath analysisfocus on volatile organic compounds. *Clinica Chimica Acta* **347**, 25–39 (2004).

[5] Wang, C. *et al.* Volatile organic metabolites identify patients with breast cancer, cyclomastopathy, and mammary gland fibroma. *Sci. Rep.* **4**, 5383 (2014).

[6] Wang, C. *et al.* Exhaled volatile organic compounds as lung cancer biomarkers during one-lung ventilation. *Sci. Rep.* **4**, 7312 (2014).

[7] Blanchet, L. *et al.* Factors that influence the volatile organic compound content in human breath. *J. Breath Res.* **11**, 016013 (2017).

[8] Wallner-Liebmann, S. *et al.* Individual human metabolic phenotype analyzed by 1h nmr of saliva samples. *J. Proteome Res.* **15**, 1787–1793 (2016).

[9] Assfalg, M. *et al.* Evidence of different metabolic phenotypes in humans. *Proc. Nat. Acad. Sci.* **105**, 1420–1424 (2008).

[10] Wald, N. J., Idle, M., Boreham, J. & Bailey, A. Carbon monoxide in breath in relation to smoking and carboxyhaemoglobin levels. *Thorax* **36**, 366–369 (1981).

[11] Weaver, L. K., Howe, S., Hopkins, R. & Chan, K. J. Carboxyhemoglobin half-life in carbon monoxide-poisoned patients treated with 100% oxygen at atmospheric pressure. *Chest* **117**, 801 – 808 (2000).
